# Potential of Mitochondria-Targeted Antioxidants to Prevent Oxidative Stress in Pancreatic *β*-cells

**DOI:** 10.1155/2019/1826303

**Published:** 2019-05-21

**Authors:** Lydie Plecitá-Hlavatá, Hana Engstová, Jan Ježek, Blanka Holendová, Jan Tauber, Lucie Petrásková, Vladimír Křen, Petr Ježek

**Affiliations:** ^1^Department of Mitochondrial Physiology, No. 75, Institute of Physiology of the Czech Academy of Sciences, Vídeňská 1083, Prague 14220, Czech Republic; ^2^Laboratory of Biotransformation, Institute of Microbiology of the Czech Academy of Sciences, Vídeňská 1083, Prague 14220, Czech Republic

## Abstract

Pancreatic *β*-cells are vulnerable to oxidative stress due to their low content of redox buffers, such as glutathione, but possess a rich content of thioredoxin, peroxiredoxin, and other proteins capable of redox relay, transferring redox signaling. Consequently, it may be predicted that cytosolic antioxidants could interfere with the cytosolic redox signaling and should not be recommended for any potential therapy. In contrast, mitochondrial matrix-targeted antioxidants could prevent the primary oxidative stress arising from the primary superoxide sources within the mitochondrial matrix, such as at the flavin (I_F_) and ubiquinone (I_Q_) sites of superoxide formation within respiratory chain complex I and the outer ubiquinone site (III_Q_) of complex III. Therefore, using time-resolved confocal fluorescence monitoring with MitoSOX Red, we investigated various effects of mitochondria-targeted antioxidants in model pancreatic *β*-cells (insulinoma INS-1E cells) and pancreatic islets. Both SkQ1 (a mitochondria-targeted plastoquinone) and a suppressor of complex III site Q electron leak (S3QEL) prevented superoxide production released to the mitochondrial matrix in INS-1E cells with stimulatory glucose, where SkQ1 also exhibited an antioxidant role for UCP2-silenced cells. SkQ1 acted similarly at nonstimulatory glucose but not in UCP2-silenced cells. Thus, UCP2 can facilitate the antioxidant mechanism based on SkQ1^+^ fatty acid anion^−^ pairing. The elevated superoxide formation induced by antimycin *A* was largely prevented by S3QEL, and that induced by rotenone was decreased by SkQ1 and S3QEL and slightly by S1QEL, acting at complex I site Q. Similar results were obtained with the MitoB probe, for the LC-MS-based assessment of the 4 hr accumulation of reactive oxygen species within the mitochondrial matrix but for isolated pancreatic islets. For 2 hr INS-1E incubations, some samples were influenced by the cell death during the experiment. Due to the frequent dependency of antioxidant effects on metabolic modes, we suggest a potential use of mitochondria-targeted antioxidants for the treatment of prediabetic states after cautious nutrition-controlled tests. Their targeted delivery might eventually attenuate the vicious spiral leading to type 2 diabetes.

## 1. Introduction

Type 2 diabetes is characterized by the frequently missing first phase of insulin secretion by pancreatic *β*-cells, which begins in the prediabetic stage, whereas the second phase of insulin secretion may be prolonged or also impaired [1–4]. At the onset of the disease, insufficiently functional *β*-cells do not meet the altered glucotoxic and enhanced metabolic demand. The deterioration of *β*-cells and changes in pancreatic islets, stemming from the pathology-induced dedifferentiation and transdifferentiation of *β*-cells, have been recently recognized as major contributors to type 2 diabetes etiology. These effectors act in parallel with the typical development of insulin resistance in peripheral tissues, originating from a metabolic, i.e., overnutrition-induced, inflammatory component [4–6].

One of the common denominators of *β*-cell deterioration and inflammation is oxidative stress [4, 7, 8]. Due to impaired autophagy, oxidized cell constituents are not cleared away, which has serious consequences for cells [9, 10]. Oxidative stress in a cell is developed with a diminished amount or function of redox buffers and antioxidant enzymes or small antioxidant molecules, which decreases the ability to detoxify the produced ROS. Oxidative stress is usually irreversible, when biological constituents are heavily affected and impaired. This may also lead to the induction of programmed cell death such as apoptosis [4–8]. In contrast, a mild oxidative stress can be reversed. There should be a detectable transient oxidative stress from the ROS burst. This burst then represents a redox signal [4].

Pancreatic *β*-cells contain relatively small amounts of redox buffers such as glutathione in their organelles and cytosolic milieu, when compared with the other cell types [11–13]. In contrast, they possess sufficient redox relaying systems, which provide redox signal propagation, besides a simple maintenance of the redox homeostasis [4, 14–16]. They may propagate redox signals from the ROS sources to the targets, thus providing redox regulations [17, 18]. A lower level of glutathione (redox buffers) enables a rather weak ROS burst of mitochondrial or cytosolic origin to rapidly propagate via the redox relaying systems to the final targets [17]. Due to the high proportion of mitochondrial network volume to cell volume [19], numerous sites of mitochondrial superoxide formation substantially contribute to the overall ROS homeostasis in pancreatic *β*-cells [7, 20, 21]. Superoxide is converted to H_2_O_2_ by dismutation, catalyzed by mitochondrial superoxide dismutases, namely, by the matrix MnSOD (SOD2) and CuZnSOD (SOD1), residing in the intermembrane/intracristal space [17, 22, 23]. Due to a relatively rapid H_2_O_2_ diffusion across the biological membranes, mitochondrial and cytosolic ROS homeostases mutually influence each other [17]. In general, antioxidants can act either (i) as direct scavengers of superoxide (or other individual ROS) or (ii) as blockers (inhibitors) of the primary superoxide formation (other individual ROS species formation) at the specific sites. Here, we deal with the antioxidants of the category (ii), i.e., with blockers of the formation of the primary superoxide at specific mitochondrial sites. Initial compounds were based on positively charged triphenylphosphonium (TPP^+^) attached to ubiquinone, such as MitoQ_10_ [24–29], or plastoquinone, such as SkQ1 [30–36]. The positive charge in TPP^+^ is sheltered by the bulky phenyl groups that enable penetration across the membrane. Since respiring mitochondria possess the negatively charged surface of the matrix phospholipid leaflet of the inner membrane (intracristal membrane), TPP^+^ penetrates readily with its “cargo,” i.e., the attached antioxidant compound via a specific hydrophobic linker.

Also, an enhanced antioxidant capacity within the matrix may be effectively transferred to the cytosol as well, where this may exert the antioxidant effect in the cytosol, despite the antioxidant agents being located in the mitochondrial matrix [17, 22, 24, 25]. This principle can be applied to the action of antioxidants targeted to the mitochondrial matrix [24–40]: when the cytosolic antioxidant mechanisms and redox buffers do not need to deal with the excessive ROS released from mitochondria to the cytosol, they have a spare capacity, which can produce a stronger antioxidant action in the cell cytosol.

In recent years, other compounds, antioxidants or other agents, are being developed and tested [36–40], as well as compounds enabling the *in vivo* assessment of mitochondrial oxidative stress [41–44]. The targeting of agents affecting redox homeostasis to the mitochondrial matrix has become rather popular and frequently studied. A plethora of compounds has been developed and tested. Besides the initial compounds, such as MitoQ_10_ [24–29] and SkQ1 [30–36], some novel compounds [36–40] are being studied, and several of them have reached the level of clinical trials.

The mitochondria-targeted antioxidants of type (ii) (blockers of sources) typically interfere with the sites of superoxide formation there but do not extensively influence the primary ROS formation or redox regulations within the cytosol [17, 22, 24, 25, 34].

Their typical action is to prevent an electron leak from the specific site to oxygen, thus preventing superoxide formation. Instead, electrons originating from a particular site are transferred to the active antioxidant moiety. When single-electron transfer takes place, the oxidized form of an antioxidant (AntOx) becomes a radical AntOx^·^. When two-electron transfer is possible, the oxidized antioxidant AntOx is thus reduced to AntOXH_2_. Nevertheless, when these products are stable and cannot be converted back to AntOx, the pool of oxidized AntOx is rapidly depleted, especially at low AntOx concentrations. The advantage of the developed antioxidants lies in their ability to be regenerated, mostly in neighbouring or distant sites that are able to neutralize the radical AntOx^·^ form or oxidize AntOXH_2_ back to AntOx. Having this property, they act at very low, typically nanomolar extracellular concentrations. Note that due to the ~180 mV electrical potential component at the inner mitochondrial membrane potential (negative inside at the matrix lipid bilayer leaflet) and ~60 mV plasma membrane potential (negative at the cytosolic side), the distribution of positively charged antioxidants can be 1 : 10,000 in favour of the matrix. Thus, a 1 nmol·l^−1^ extracellular AntOx concentration becomes 10 *μ*mol·l^−1^ relative to the matrix volume. Moreover, due to its hydrophobic character and consequent compartmentalization within the tiny inner mitochondrial membrane lipid bilayer volume, the effective concentration within the lipid bilayer can reach up to 10 mmol·l^−1^.

The disadvantage of the above regeneration of the reduced (radical) antioxidant species lies in the possibility of instead exhibiting prooxidative properties [28, 34]. Thus, the balance between the electron scavenging and regenerating reaction (AntOx→AntOXH_2_ and AntOXH_2_→AntOx) can decide, whether the compound will have antioxidant or prooxidant properties. In particular, when regeneration takes place at a distinct site from that of AntOX reduction, the antioxidant/prooxidant prevalence can depend on the metabolic state or mode. For example, mitochondria-targeted antioxidant MitoQ_10_ exerts a prooxidant effect, by effectively retarding the ubiquinone-mediated electron transfer between the respiratory chain complexes I, II, and III within cells under physiological conditions [28]. In contrast, MitoQ_10_ is able to bridge or bypass the locus of the electron transfer retardation in the pathologically affected complex I. This can be simulated by the ability of MitoQ_10_ to suppress rotenone-induced superoxide formation [28].

**Figure 1:**
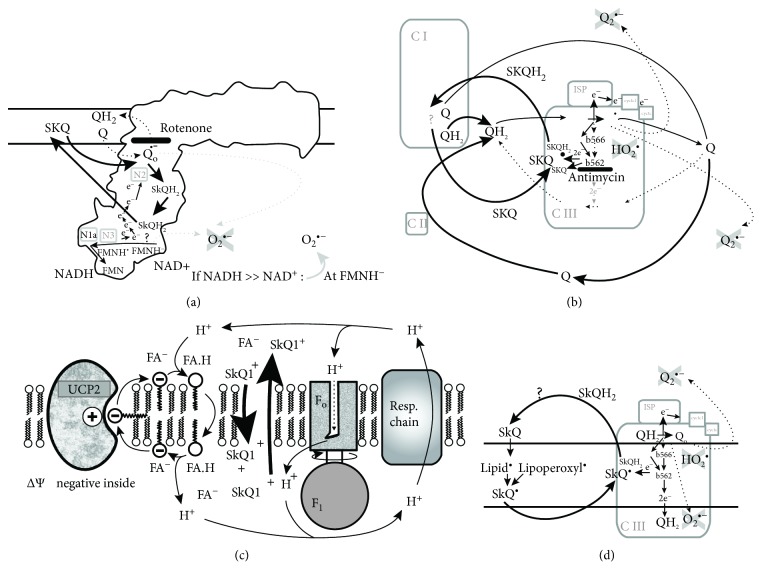
Suggested mechanisms for antioxidant action of matrix-targeted antioxidant SkQ1: (a) antioxidative two-electron reduction of SkQ1 to SkQ1H_2_ plus regeneration (oxidation of SkQ1H_2_) within the sole complex I, based on Reference [34]; (b) antioxidative two-electron reduction of SkQ1 to SkQ1H_2_ at site III_Qo_ of complex III and regeneration at the complex I, based on Reference [34]; (c) SkQ1 participation in SkQ1^+^ fatty acid anion^−^ ion pairing and consequent cycling of SkQ1 and fatty acid, based on Reference [30, 32]—this cycling partially uncouples protonic coupling in mitochondria and hence attenuates superoxide formation; (d) single-electron reduction of SkQ1 to SkQ1 radical during lipid peroxidation, based on Reference [30], with as yet unknown regeneration.

Similarly, mitochondria-targeted plastoquinone SkQ1 exerts an antioxidant action within a prooxidant milieu (rotenone-induced and/or antimycin *A*-induced superoxide formation), despite lacking the ability to suppress basal superoxide formation in glycolytic hepatocellular carcinoma HepG2 cells [34]. In this case, there is a dependence on the metabolic mode. In glycolytic hyperglycemic HepG2 cells, i.e., at 25 mM glucose, SkQ1 even exhibited a prooxidant effect in cells with no other agents added [34]. However, several mechanisms of SkQ1 antioxidant action have been reported (Figures 1(a)–1(c)).

We have previously demonstrated that oxidized SkQ1 can be reduced to SkQ1H_2_ in the vicinity of the I_Q_ site for superoxide formation within complex I of the mitochondrial respiratory chain [34]. SkQ1H_2_ is most likely regenerated (oxidized back to SkQ1) at the flavin I_F_ site of superoxide formation within complex I (Figure 1(a)). However, no antioxidant properties of SkQ1 were observed at high substrate pressure (a high NADH/NAD^+^ ratio) [34].

It has been previously recognized that superoxide is formed at the flavin I_F_ site at just such a high NADH/NAD^+^ ratio [17, 34]. Hence, we expected that such a high substrate pressure prevents SkQ1H_2_ regeneration to SkQ1. In contrast, at a medium or low NADH/NAD^+^ ratio, SkQ1 exhibited antioxidant properties, since SkQ1H_2_ was readily regenerated at the flavin I_F_ site [34].

The second mechanism, for a possible antioxidant role of SkQ1, focuses on the interference at the site III_Qo_ of the complex III [34] (Figure 1(b)). However, even in this case, SkQ1H_2_ is regenerated (oxidized to SkQ1) at the flavin I_F_ site of the complex I [34]. Moreover, a third antioxidant mechanism for SkQ1 has been suggested by Skulachev et al. [30, 32], due to its ability to uncouple proton coupling in mitochondria in the presence of a basal amount of free fatty acid (Figure 1(c)). Under certain circumstances, the uncoupling ATP synthesis from respiratory chain proton pumping (i.e., proton short circuiting) may have an attenuating effect on mitochondrial superoxide formation [17, 21, 22]. Skulachev and colleagues have also suggested that an ion pair of SkQ1 with anionic fatty acid^−^ is able to penetrate the lipid bilayer membrane [30, 32]. Note that SkQ1 is typically positively charged as SkQ1^+^. Since positively charged SkQ1^+^ also crosses the lipid bilayer, the neutral fatty acid flip-flop carries a proton from the outer (intracristal space) inner membrane leaflet back to the mitochondrial matrix (internal) inner membrane leaflet and thus causes uncoupling (a proton short circuit) [30, 32].

Finally, a fourth antioxidant mechanism has been suggested for SkQ1, consisting of the formation of the SkQ1^·^ radical, where SkQ1 interferes with the chain reaction of lipid peroxidation (Figure 1(d)) [30]. Despite the benefits observed *in vivo* during preclinical and clinical testing of SkQ1 and its derivatives, a complex pattern of behaviour can be expected depending on the cell type and metabolic mode.

This is why more specific antioxidant agents have been developed. Recently, Brand and colleagues and Wong et al. have developed mitochondria-targeted antioxidants acting at the specific sites of superoxide formation [45–47]. For example, the suppressor of complex I site Q electron leak (S1QEL) acts at the ubiquinone (coenzyme Q) site I_Q_ of superoxide formation within complex I [45–47]. This site is known to produce superoxide during the reverse electron transfer occurring after, e.g., succinate accumulation, in cardiomyocytes during ischemia, while the concomitant superoxide burst is the primary damaging agent upon reperfusion in ischemic heart disease [45, 48]. Similar mechanisms stemming from succinate accumulation have been reported recently for other tissues as well [49]. Also, the suppressor of complex III site Q electron leak (S3QEL) has been determined by a chemical screen to act at the outer ubiquinone site III_Qo_ of superoxide formation within complex III (i.e., located on the inner membrane surface oriented toward the intracristal space) [45–47]. Site III_Qo_ typically plays an important role within the Q cycle of mitochondrial electron transfer. The effective retardation of the Q cycle, e.g., by slowing down the cytochrome *c* turnover, results in enhanced superoxide formation within this site [17]. For example, this can be simulated by the addition of antimycin *A* [34]. Being aware of the complexity of mitochondria-targeted antioxidant roles, in this work, we tested the basic antioxidant properties of three mitochondria-targeted antioxidants, SkQ1, S1QEL, and S3QEL, in a model of pancreatic *β*-cells, INS-1E cells, and in isolated pancreatic islets. We demonstrate that only SkQ1 and S3QEL exhibit the prevailing antioxidant role in INS-1E cells and discuss why the antioxidant role of these compounds depends on specific metabolic conditions. On this basis, we predict which compounds might be suitable for retarding or curing the oxidative stress component in the development of type 2 diabetes.

## 2. Materials and Methods

### 2.1. Materials

Reagents were from Sigma-Aldrich (St. Louis, MO), unless stated otherwise. SkQ1 was kindly provided by Prof. Vladimir Skulachev (Moscov State University, Russia). S1QEL and S3QEL were from Life Chemicals (shipped from Spoluka Chemical Company, Kiev, Ukraine).

### 2.2. Cell Cultures

Rat insulinoma INS-1E cells (C0018009, AddexBio, San Diego, CA) were cultured in 11 mmol·l^−1^ glucose and RPMI 1640 medium supplemented with 5% (*v*/*v*) fetal calf serum, 10 mmol·l^−1^ HEPES, 1 mmol^−1^ pyruvate, 50 *μ*mol·l^−1^ mercaptoethanol, 50 IU/ml penicillin, and 50 *μ*g/ml streptomycin [50, 51]. Routine 1-hour, 2-hour, or 15-hour incubations were performed with 3 mmol·l^−1^ glucose to lower glucose and diminish beneficial autocrine effects [50, 51].

### 2.3. Mouse Pancreatic Islet Isolation and Culturing

Experiments with mice (C57Bl/6J strain, The Jackson Laboratory, Bar Harbor, MN) were approved by the Animal Care and Use Committee (Inst. Molecular Genetics, ASCR) in accordance with the European Union Directive 2010/63/EU for animal experiments, U.K. Animals (Scientific Procedures) Act, 1986, and the Guide for the Care and Use of Laboratory Animals (NIH Publication No. 85-23, revised 1996) and the ARRIVE guidelines. Mice were anesthetized using a mixed solution of Zoletil (40 mg/kg, Virbac SA, Carros, France) and 2% Rometar (10 mg/kg, Spofa, Czech Republic). Pancreases were perfused with collagenase IX (Sigma-Aldrich) solution in HBSS buffer and trimmed with surgical scissors. The pancreases were subsequently digested with collagenase for 10 min at 37°C. To remove exogenous tissue, samples were washed with HBSS two times. The tissue was then filtered through a 500 *μ*m cell strainer, and islets were separated on a Ficoll gradient (Sigma-Aldrich) by centrifugation. The islets were placed in CMRL medium (PAN-Biotech, Aidenbach, Germany) and kept overnight at 37°C. The next day, the islets were seeded on wells coated with Biolaminin (BioLamina, Sundyberg, Sweden) and incubated overnight at 37°C. Experiments were carried out on the third day.

### 2.4. UCP2 Silencing

This study uses the same silencing protocol as that developed by Ježek et al. [51], and the description below partly reproduces their wording. A BLOCK-iT Pol II miR RNAi system (Thermo Fisher Scientific, Waltham, MA; formerly Life Technologies) served to express miRNAs against rat *Ucp2*. The two rat miRNA sequences were designed as follows: 5′-TACAGAGTCGTAGAGGCCAATGTTTTGGCCACTGACTGACATTGGCCTACGACTCTGTA-3′ and 5′-ATTTCGGGCAACATTGGGAGAGTTTTGGCCACTGACTGACTCTCCCAATTGCC CGAAAT-3′, using the BLOCK-iT RNAi Designer and annealed into double-strand oligonucleotides. They were inserted into the linearized miRNA expression vector pcDNA6.2-GW/EmGFP-miR and were chained up into tandem constructs. Vectors containing UCP2-miRNA or scrambled miRNA were cloned by Gateway BP/LR reaction into the pLenti6.2/V5-DEST expression vector. Final constructs were validated by sequencing. Lentiviral expression plasmids were cotransfected with ViraPower Packaging Mix (Thermo Fisher) into 293LTV cells using Lipofectamine 2000 (Thermo Fisher). The lentiviral stock was used to transfect INS-1E cells, followed by selection of a stably transduced cell line by blasticidin and verification by a green fluorescent protein (GFP) inherent cytosolic reporter.

### 2.5. Confocal Microscopy Monitoring of Superoxide Released into the Mitochondrial Matrix

Monitoring was performed in a Leica TCS SP2 AOBS or alternatively Leica TCS SP8 confocal microscopy systems. A triphenylphosphonium-conjugated dihydroethidine, MitoSOX Red (Thermo Fisher), was used to monitor the rates (*J*_m_) of *in situ* surplus superoxide release into the mitochondrial matrix [34, 51, 52]. The surplus represents the portion of superoxide not neutralized by the matrix MnSOD. This study uses the method progressively developed by Dlasková et al. [52] and Ježek et al. [34, 51], and the method description partly reproduces their wording [34, 51, 52].

While using rates, any variations in background nonspecific fluorescence are eliminated, and the method was previously found to be feasible for the semiquantification of matrix-released superoxide even at low or collapsed mitochondrial inner membrane electric potential ΔΨ_m_, due to the intercalation of MitoSOX Red into the mitochondrial DNA [52]. Also, MitoSOX Red insulation from the cytosolic events has been demonstrated by its insensitivity to externally added *tert*-butylhydroperoxide [51].

The excitation used was at 514 nm with a 20 mW Argon laser, with emission collected between 550 and 650 nm. INS-1E cells were loaded with 4 *μ*mol·l^−1^ MitoSOX Red for 15 min. A series of confocal images were usually taken every 30 s for 20 min. Regions of interest corresponding to mitochondria were selected using the software Ellipse (ViDiTo, Košice, Slovakia). Changes in integrated fluorescence intensity were quantified from plots of fluorescence in the selected areas vs. time, providing integral rates *J*_m_.

### 2.6. Liquid Chromathography-Mass Spectroscopy-Based Assay for Quantification of Mitochondrial ROS Accumulation Using the MitoB Probe

We employed the matrix-targeted H_2_O_2_-specific probe MitoB, in order to quantify the accumulated H_2_O_2_ formed during the chosen time interval in the mitochondrial matrix [41]. The method originally derived by Murphy and colleagues [41–44] was adopted accordingly. The boron-containing MitoB compound diffuses freely to the mitochondrial matrix, where it is oxidized by H_2_O_2_ to the MitoP compound. Equilibrium between the concentrations of both compounds in the matrix and the culture medium is readily established. As a result, this setup enables estimations of the relative amounts of MitoP and MitoB externally in the culture medium, without the requirement to assay it within the matrix compartment. These species are therefore quantified by LC-MS after sampling during the period of mitochondrial matrix H_2_O_2_ accumulation. The resulting calculated MitoP/(MitoB+MitoP) ratios are proportional to the amount of H_2_O_2_ accumulated in the given time periods.

The INS-1E cells were grown under standard conditions as described above. Prior to the experiment, cells were preincubated in the culturing medium with 3 mmol·l^−1^ or 11 mmol·l^−1^ glucose, respectively, for 2 hr at 37°C. Afterwards, the medium was replaced, and the MitoB probe (5 *μ*mol·l^−1^; Sigma-Aldrich) was added, together with the given glucose concentration and selected agents, when required. The cells were subsequently incubated for another 2 hr at 37°C. After the treatment, 500 *μ*l aliquots of the medium was removed and snap-frozen on dry ice. The samples were stored at –80°C before further processing.

The experiment was also performed with isolated mouse pancreatic islets. The islets were preincubated for 2 hours at 37°C in CMRL medium (PAN-Biotech), containing 5 mmol·l^−1^ glucose. Then, the medium was replaced with the CMRL medium with either 5 or 25 mmol·l^−1^ glucose, and the MitoB probe (5 *μ*mol·l^−1^; Sigma-Aldrich) and selected agents were added. Next, the islets were incubated for 4 hours at 37°C. 200 *μ*l of medium was taken, snap-frozen on dry-ice, and stored before further processing at -80°C.

For MitoB and MitoP quantification, samples were thawed, and 200 *μ*l aliquots was used. All samples were spiked with 500 nmol·l^−1^ internal standards of d_15_-MitoB and d_15_-MitoP (Cayman Chemicals) and vortexed for 30 s. Subsequently, aliquots of 50 *μ*l of 100% acetonitrile/0.1% formic acid (vol/vol) were added, and samples were vortexed for 30 s and centrifuged for 10 min at 16,000 ×g at room temperature. Finally, 100 *μ*l sample aliquots was used for LC-MS analysis.

Mass spectra were obtained using a Shimadzu Prominence system consisting of a DGU-20A3 mobile phase degasser, two LC-20 AD solvent delivery units, a SIL-20 AC cooling auto sampler, a CTO-10AS column oven, and SPD-M20A diode array and LCMS-2020 mass detectors with a single quadrupole, equipped with an electrospray ion source (Shimadzu, Kyoto, Japan). Binary gradient elution was used as follows: mobile phase A contained water and 0.1% formic acid; mobile phase B was 100% acetonitrile. The linear gradient was used as follows: 0 min 30% B, 6 min 60% B, and 7 min 30% B, 10 min stop. The flow rate was 0.4 ml·min^−1^ at 25°C, and the injection volume was 10 *μ*l.

The MS parameters were as follows: positive mode was used; the ESI interface voltage was 4.5 kV; detector voltage was 1.15 kV; the nebulizing gas flow was 1.5 ml·min^−1^; drying gas flow was 15 ml·min^−1^; heat block temperature was 200°C; DL temperature was 250°C; SIM mode: Mito B [M+H]+ 397, Mito P [M+H]+ 369, Mito B deut. [M+H]+ 412, Mito P deut. [M+H]+ 384. The software LabSolutions version 5.75 SP2 was used. The ratio of MitoP/MitoB was estimated from the respective areas under the curve obtained by the MS analysis.

### 2.7. Three-Dimensional Imaging by Superresolution BiplaneFPALM Microscopy

This study uses a similar protocol to that developed by Plecitá-Hlavatá et al. [53], and the description below partly reproduces their wording. Wild-type tetramerizing Eos with a COX8A mitochondrial address (pwt-EosFP; MoBiTec, Rastatt, Germany) was subcloned into the pDONR221 vector (Thermo Fisher Scientific) and then into the pLenti6.3/V5-DEST vector. Lentiviral expression plasmids were cotransfected with ViraPower Packaging Mix (Thermo Fisher Scientific) into 293LTV cells using Lipofectamine 2000 (Thermo Fisher). The lentiviral stock was used to transfect INS-1E cells, which were subsequently fixed with 4% paraformaldehyde and 0.05% glutaraldehyde (both EMS, Fort Washington, PA, USA) and imaged with a BiplaneFPALM instrument (Bruker, formerly Vutara Inc., Salt Lake City, UT, USA), as described by Plecitá-Hlavatá et al. [53]. Diameter and fragmentation screening along the mitochondrial reticulum network length was performed by transferring data via an image filter with Gaussian smoothing into the software Amira 5.5 (FEI; Visualization Sciences Group, Burlington, MA, USA), using the Autoskeleton function.

### 2.8. Cell Viability Assay

The INS-1E cells were grown under standard conditions as described above. Prior to the experiment, cells were preincubated in the culturing medium with 3 mmol·l^−1^ glucose for 2 hr at 37 C. Afterwards, the medium was replaced with a fresh one, and then, the required glucose was adjusted to 25 mmol·l^−1^. Cells were subsequently incubated for another 20 min and/or 2 hr at 37°C. Only for these second 2 hr incubations, the 5 *μ*mol·l^−1^ MitoB probe was also added together with 20 *μ*mol·l^−1^ rotenone or 1 *μ*mol·l^−1^ antimycin *A* in the desired samples. After the incubation, cells were washed with PBS and treated with trypsin to detach them. Fresh medium was then added in a 1 : 1 ratio with trypsin, and 10 *μ*l aliquots was mixed with 10 *μ*l of trypan blue. The viability was then estimated using the Cell Counting Chamber and Cell Counter (Thermo Fisher Scientific).

### 2.9. Viability Assay for Pancreatic Islets

To estimate effects of antioxidants on pancreatic islet survival in culture for a given time period, the viability assay was performed directly after the MitoB assay. The islets were incubated with propidium iodide and acridine orange (Thermo Fisher) for 10 min at 37°C in the dark. These solutions were added directly to the CMRL medium according to the manufacturer's instructions. The islets were then washed once with 1x PBS to wash out the excess stain. The live (green fluorescence) and dead (red fluorescence) islets were counted manually by two independent observers under the fluorescence microscope (Olympus IX2-UCB).

### 2.10. Statistical Analysis

Where not stated otherwise, *n* = 3 biological estimates were evaluated, and error bars represent standard deviations. For most of the data sets, ANOVA was used for statistical analyses with the Tukey test on the prevalidated data through a normality test (SigmaStat 3.1, Systat Software, San Jose, CA). For comparing the two data sets, Student's *t*-tests were employed.

## 3. Results

### 3.1. Effects of Mitochondria-Targeted Plastoquinone SkQ1 on Superoxide Release to the Mitochondrial Matrix in INS-1E Cells

**Figure 2:**
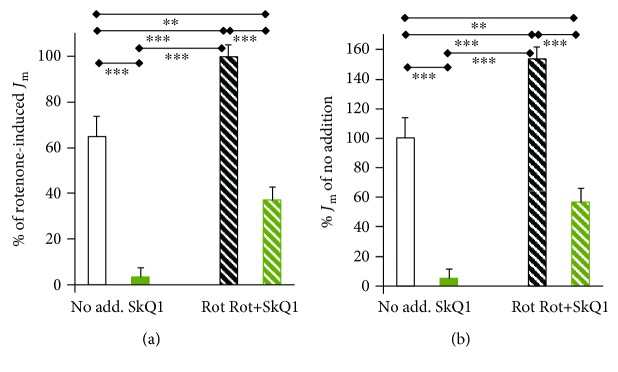
Effect of matrix-targeted antioxidant SkQ1 on MitoSOX Red responses to matrix-released superoxide. Relative rates *J*_m_ for superoxide release to the mitochondrial matrix in INS-1E cells, incubated in medium containing 25 mmol·l^−1^ glucose, are normalized (a) to the average rate *J*_m_ observed with 20 *μ*mol·l^−1^ rotenone or (b) to the average obtained in controls without any agent addition. When indicated, 1 nmol·l^−1^ SkQ1 was added at the beginning of 20 min of confocal microscopy time-lapsed monitoring. The error bars represent the standard deviations of 4–6 measurements. ANOVA (*n* = 4–6): ∗∗∗*p* < 0.001; ∗∗*p* < 0.05.

1 nmol·l^−1^ mitochondria-targeted plastoquinone SkQ1 nearly completely suppressed the superoxide release rates *J*_m_ to the mitochondrial matrix in intact INS-1E cells (Figures 2(a) and 2(b)). When 20 *μ*mol·l^−1^ rotenone was added, the superoxide release rates (*J*_m_) nearly doubled (Figures 2(a) and 2(b)). At 1 nmol·l^−1^, SkQ1 restricted these rotenone-induced *J*_m_ rates with 25 mmol·l^−1^glucose by about two-thirds (Figure 2(a)). We normalized the measured rates to the average *J*_m_ rate obtained either with rotenone (Figure 2(a)) or with no agents (Figure 2(b)).

**Figure 3:**
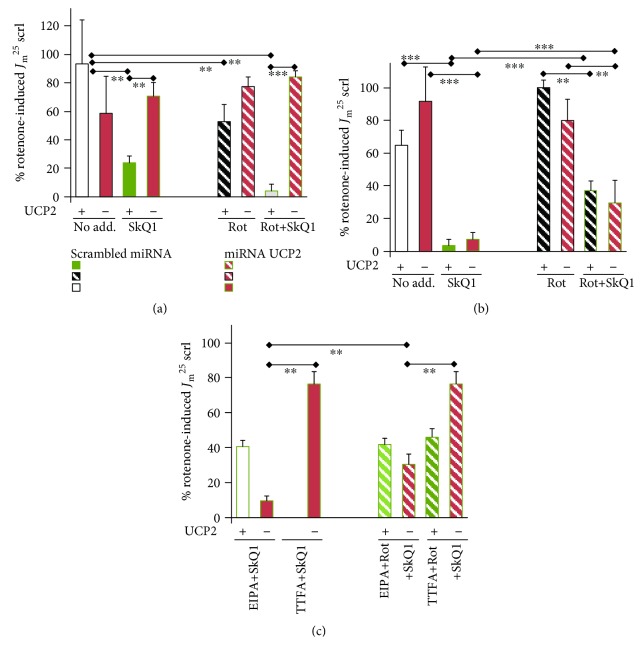
Effect of mitochondria-targeted antioxidant SkQ1 on MitoSOX responses to mitochondrial matrix-released superoxide. *Color code*: *all red or red stripes (even bars)*: UCP2-silenced INS-1E cells; *other (odd bars)*: INS-1E cells transfected with miRNA of a scrambled sequence; *green or green-edge bars*: 1 nmol·l^−1^ SkQ1; *downward diagonal stripes*: 20 *μ*mol·l^−1^ rotenone. Relative rates for superoxide release to the mitochondrial matrix *J*_m_ in INS-1E cells were normalized to those *J*_m_ rates induced by rotenone at 25 mmol·l^−1^ glucose in “control” cells transfected with scrambled miRNA. Agents were added at the beginning of 20 min confocal microscopy time-lapsed monitoring. Data in (c) were measured in the presence of 1 *μ*mol·l^−1^ EIPA or 40 *μ*mol·l^−1^ TTFA as indicated. The error bars represent the standard deviation of 3–9 measurements. ANOVA in (a), (b) *n* = 3– 9, and (c) *n* = 3–5: ∗∗∗*p* < 0.001; ∗∗*p* < 0.05.

In order to demonstrate whether part of the SkQ1 antioxidant mechanism can originate from the SkQ1 cycling with ion-paired fatty acids, as suggested by Skulachev et al. [30, 32] (Figure 1(c)), we silenced the mitochondrial uncoupling protein UCP2 in INS-1E cells (Figures 3(a) and 3(b)). UCP2 mediates a uniport of anionic fatty acids [51, 54]. However, UCP2 better utilizes nascent fatty acids just immediately after their cleavage from phospholipids by mitochondrial phospholipase A2 isoform *γ* [51]. Moreover, without redox activation of this phospholipase, UCP2 is largely inactive [51]. Nevertheless, when UCP2 is functional in INS-1E cells, it attenuates the mitochondrial superoxide production [51]. This may explain the observed higher superoxide release rates *J*_m_ upon UCP2 silencing (Figure 3(b)). The transport across the inner membrane lipid bilayer of the ion pair {SkQ1^+^and fatty acid anion^−^} suggested by Skulachev et al. [30, 32] can be concurrent to the UCP2-mediated fatty acid cycling. Hence, we investigated whether SkQ1 antioxidant action is modulated upon UCP2 silencing, for which we employed lentiviral transfection with miRNAs, as previously described in [51].

Again, we normalized all measured rates to the average rotenone-induced *J*_m_ rate obtained with 25 mmol·l^−1^ glucose in cells transfected with scrambled miRNA (*J*_m_^25^scrl). Thus, with 25 mmol·l^−1^ glucose, SkQ1 yielded a similar antioxidant pattern in control cells, transfected with the miRNA containing the scrambled sequence (ntg cells), as well as in cells with silenced UCP2 (Figure 3(b)). With 3 mmol·l^−1^ glucose, SkQ1 exhibited the antioxidant effect, though at a lower intensity in cells with no agents added (Figure 3(a)). This may stem from the fact that the mitochondrial matrix superoxide release is faster at a low glucose concentration (cf. first bars in Figures 3(a) and 3(b)). At this low glucose level, SkQ1 strongly inhibited the rotenone-induced *J*_m_ rates. However, the rotenone-induced *J*_m_ rates of matrix superoxide release were lower than without any agent added. The latter indicates the participation of a reverse electron transfer component.

In contrast, no antioxidant effect of SkQ1 was found upon UCP2 silencing (Figure 3(a)). This resembles a phenomenon reported by Skulachev et al. [30], in which the carboxyatractyloside blockage of the mitochondrial ADP/ATP carrier decreased the {SkQ1^+^and fatty acid anion^−^} ion-paired cycling and hence prevented the attenuation of mitochondrial ROS formation [30]. As a result, ROS formation was high. Note that the UCP2, as well as the ADP/ATP carrier (also termed the adenine nucleotide transporter), possess the ability to mediate the uniport of anionic fatty acids [30, 54]. We interpret the obtained data by the ability of UCP2 to substitute for the ADP/ATP carrier in facilitating cycling involving {SkQ1^+^and fatty acid anion^−^}. This mechanism (Figure 1(c)) may be dominant at the low 3 mmol·l^−1^ glucose level due to the high NADH/NAD^+^ ratio (termed substrate pressure) imposed on complex I in INS-1E cells [4].

We also tested the influence of proton pumping inhibition at complex I by the hydrophobic amiloride derivative, 5-(*N*-ethyl-*N*-isopropyl) amiloride (EIPA), and the effect of complex II inhibition by thenoyltrifluoroacetone (TTFA). Note that EIPA has been previously reported to enhance superoxide formation within complex I by the inhibition of proton pumping [52]. EIPA slightly inhibited the antioxidant effect of 1 nmol·l^−1^ SkQ1, independent of the presence or absence of UCP2 (cf. Figures 3(c) and 3(b)). TTFA, as the complex II inhibitor, interferes with the membrane pool of ubiquinone [28]. This retards the electron transfer via the respiratory chain independent of the I_F_ and I_Q_ sites as well as the III_Qo_ site. Hence, TTFA should prevent the SkQ1 antioxidant role, and this was in fact observed. Thus, TTFA eliminated the sole antioxidant effect of 1 nmol·l^−1^ SkQ1 in UCP2-silenced INS-1E cells at 25 mmol·l^−1^ glucose (Figure 3(c)). With rotenone, however, neither EIPA nor TTFA influenced the partial antioxidant effect of SkQ1 in ntg cells, having intact UCP2. This is because SkQ1 reduction/oxidation takes place at the I_Q_/I_F_ sites of complex I, and therefore, the SkQ1 antioxidant ability is independent of complex II [34]. Likewise, EIPA did not affect the SkQ1 antioxidant intensity even in the UCP2-silenced cells, but TTFA decreased it (cf. Figures 3(c) and 3(b)). In the latter case, a mild uncoupling not only short circuits the proton pumping of complex I but also acts in complexes III and IV. Since complex III receives ubiquinol from complex II, its inhibition causes the observed effect.

### 3.2. Effects of Mitochondrial Matrix-Targeted Antioxidants S1QEL and S3QEL on Superoxide Release into the Mitochondrial Matrix in INS-1E Cells

**Figure 4:**
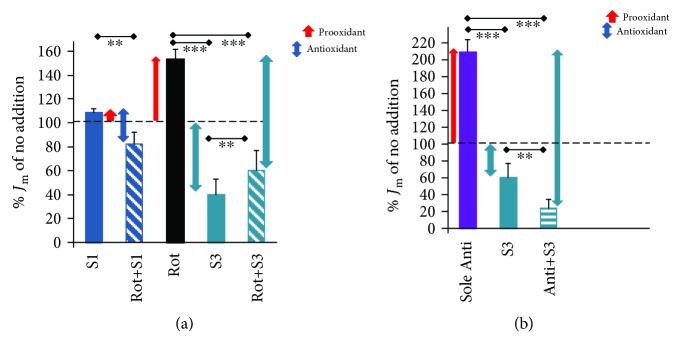
Effect of matrix-targeted antioxidants S1QEL and S3QEL on MitoSOX Red responses to matrix-released superoxide. Relative rates *J*_m_ for superoxide release to the mitochondrial matrix *J*_m_ in INS-1E cells, incubated in a medium with 25 mmol·l^−1^ glucose, are normalized to those obtained in controls without any agent addition ((a, b); dashed line). When indicated, also 10 *μ*mol·l^−1^ S1QEL (S1) or S3QEL (S3) without or together with 1 *μ*mol·l^−1^ antimycin *A* (Anti) or 20 *μ*mol·l^−1^ rotenone (Rot) was added at the beginning of 20 min confocal microscopy time-lapsed monitoring. The error bars represent the standard deviation of 4–6 measurements (12 for sole S1QEL). ANOVA (*n* = 4–6): ∗∗∗*p* < 0.001; ∗∗*p* < 0.05.

The suppressor of the electron leak targeted to the specific ubiquinone site I_Q_ of complex I, S1QEL, exhibited a slight prooxidant effect, when added to intact INS-1E cells with 25 mmol·l^−1^ glucose (Figure 4(a)). However, S1QEL only slightly decreased the rotenone-induced superoxide release *J*_m_ rates (Figure 4(a)). Antimycin *A* enhanced rates *J*_m_ up to 2-fold when normalized to regular *J*_m_ rates at 25 mmol·l^−1^ glucose and no agents added (Figure 4(b)). For this experiment, INS-1E cells were also incubated with 25 mmol·l^−1^ glucose. The suppressor of the electron leak targeted to the specific quinone site III_Qo_ of complex III, S3QEL, prevented up to 90% of the antimycin *A*-induced mitochondrial matrix superoxide release rates (Figures 4(a) and 4(b)). Interestingly, S3QEL also suppressed about 60% of the rotenone-induced matrix superoxide release rates (Figures 4(a) and 4(b)). This effect may be explained by the retardation of the ubiquinol/ubiquinone shuttle, i.e., Q shuttle, between complex I and complex III, in which the complex III electron transfer (Q cycle) is nearly completely blocked.

### 3.3. Cell Viability after Rotenone or Antimycin *A* Incubations

**Figure 5:**
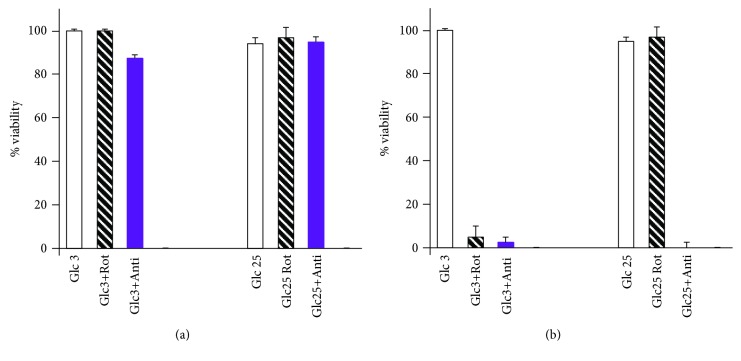
INS-1E cell viability after 20 min or 2 hr incubations. Cell viability was estimated in 3 independent experiments and is expressed in % of viable cells after 20 min (a) and 2 hr (b) incubations, either with no agents added or with 20 *μ*mol·l^−1^ rotenone (+Rot) or 1 *μ*mol·l^−1^ antimycin *A* (+Anti), in a medium with 3 mmol·l^−1^ glucose (Glc3) or 25 mmol·l^−1^ glucose (Glc25). The error bars represent the standard deviation of 3 measurements.

Next, we checked the viability of INS-1E cells after the required time intervals for MitoSOX Red monitoring (20 min) or the MitoB assessment of ROS accumulation (2 hr; here, MitoB was also present during such incubation). With both 3 mmol·l^−1^ and 25 mmol·l^−1^ glucose, INS-1E cells survived after 20 min in the presence of 20 *μ*mol·l^−1^ rotenone or 1 *μ*mol·l^−1^ antimycin *A* (Figure 5(a)). Despite the complex I inhibition by rotenone, cells still respired, though by much lower rates, due to succinate utilization by complex II (succinate dehydrogenase) of the respiratory chain. Although a drastic respiration decrease occurred with 1 *μ*mol·l^−1^ antimycin *A*, the 20 min time interval was not sufficient to complete cell death. In light of these observations, the data of Figure 4 were thus validated as representing the antioxidant effects unbiased by the decrease in cell viability. In contrast, 2 hr incubations with either 20 *μ*mol·l^−1^ rotenone or 1 *μ*mol·l^−1^ antimycin *A* led to a profound cell death at 3 mmol·l^−1^ glucose (Figure 5(b)). After 2 hr incubations, INS-1E cells were still satisfactorily viable in the presence of 20 *μ*mol·l^−1^ rotenone at 25 mmol·l^−1^ glucose but not with antimycin *A* (Figure 5(b)). This fact only enables a valid MitoB 2 hr lasting assay for the rotenone and 25 mmol·l^−1^ glucose.

### 3.4. Cell Respiration Induced by Palmitic Acid

**Figure 6:**
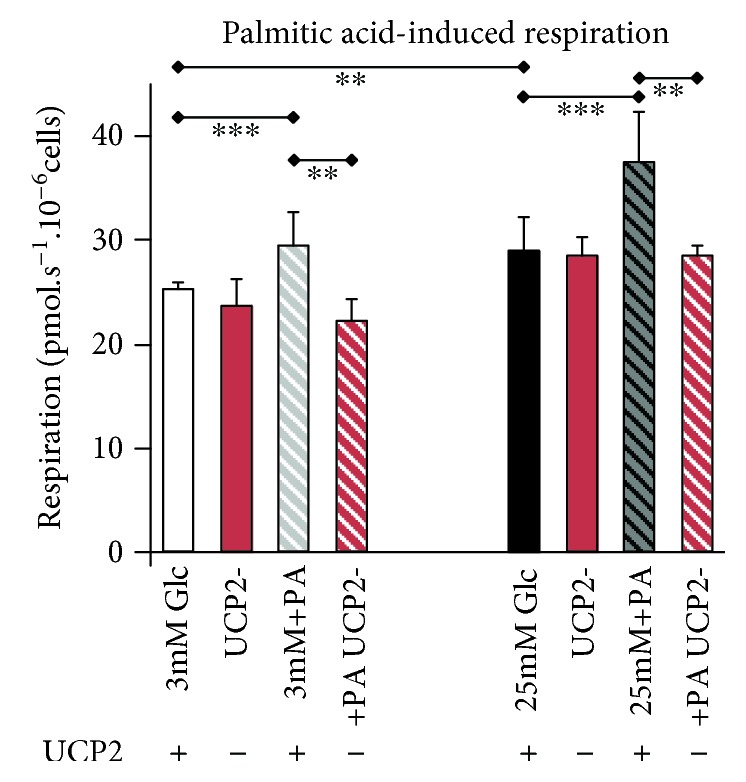
Respiration induced by palmitic acid in nontransgenic *vs*. UCP2-silenced INS-1E cells. Respiration was estimated in 10–14 and 3 independent experiments for nontransgenic (ntg) and UCP2-silenced INS-1E cells. The ntg cells were transfected with miRNA of a scrambled sequence. When indicated (+PA), palmitic acid in bovine serum albumin solution was added to reach a total amount of 75 nmol palmitic acid *per* l0^6^ cells. As indicated, either 3 mmol·l^−1^ glucose (Glc3) or 25 mmol·l^−1^ glucose (Glc25) was present in the assay medium. The error bars represent the standard deviations. ANOVA: ∗∗∗*p* < 0.001; ∗∗*p* < 0.05.

We also performed another control, related mainly to the data of Figure 3. We demonstrated that fatty acids, such as palmitic acid (in a total amount of 75 nmol palmitic acid per l0^6^ cells), are able to partially uncouple the respiration of INS-1E cells and that this ability ceases upon UCP2 silencing (Figure 6). Palmitic acid-induced uncoupling was manifested as a significant elevation of cell respiration, which was not observed in UCP2-silenced INS-1E cells. Despite endogenous fatty acids being much lower in the experiments of Figure 3, we demonstrated an ability of UCP2 in INS-1E cells to mediate palmitic acid-induced uncoupling. For more detailed information, see Reference [51].

### 3.5. MitoB LC-MS-Assisted Quantification of Accumulated ROS vs. Effects of Mitochondria-Targeted Antioxidant Agents

**Figure 7:**
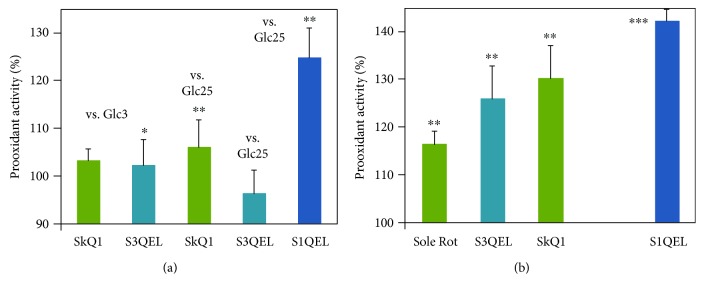
INS-1E cells: prooxidant or antioxidant effects of matrix-targeted antioxidants SkQ1, S1QEL, and S3QEL on natural or rotenone-induced 2 hr ROS accumulation estimated by MitoB. Prooxidant activity (>100%) or antioxidant activity (<100%) is related to ROS accumulated after 2 hr in INS-1E cells. Normalization, i.e., 100%, was always taken to be the measurement with no other agent added at the indicated glucose concentration. INS-1E cells were incubated in a medium with 3 mmol·l^−1^ glucose (vs. Glc3) or 25 mmol·l^−1^ glucose (vs. Glc25) without (a) or with 20 *μ*mol·l^−1^ rotenone (b). When indicated, 1 nmol·l^−1^ SkQ1, 10 *μ*mol·l^−1^ S1QEL, or S3QEL were added at the beginning. The error bars represent the standard deviation of 3 measurements. Student's *t*-test gave statistical significance where indicated as follows: ∗∗*p* < 0.05; ∗*p* < 0.001.

**Figure 8:**
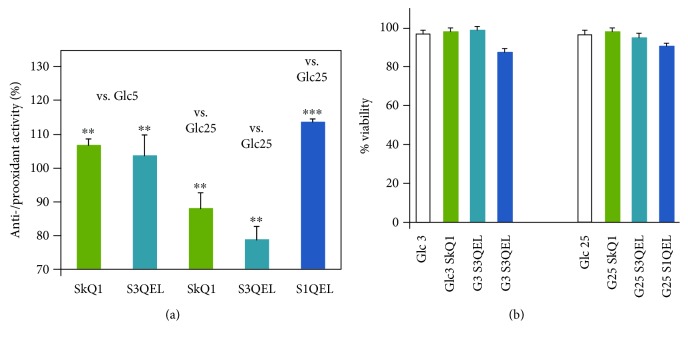
Isolated pancreatic islets: (a) prooxidant effects or antioxidant effects of matrix-targeted antioxidants SkQ1, S1QEL, and S3QEL on natural 4 hr ROS accumulation estimated by MitoB; (b) viability after 4 hr. Prooxidant activity (>100%) or antioxidant (<100%) activity is related to ROS accumulated after 4 hr in isolated pancreatic islets. Normalization, i.e., 100%, was taken to be the measurement with no agent added (a) or as % of live cells (b). Medium either contained 5 mmol·l^−1^ glucose (vs. Glc5), nonstimulating with regard to insulin secretion or 25 mmol·l^−1^ glucose (vs. Glc25), stimulating insulin secretion. Where indicated, 1 nmol·l^−1^ SkQ1, 10 *μ*mol·l^−1^ S1QEL, or S3QEL were added at the beginning of 4 hr incubations. The error bars represent the standard deviation of 3 measurement. Student's *t*-test gave statistical significance where indicated as follows: ∗∗*p* < 0.05; ∗∗∗*p* < 0.001.

As stated above, 2 hr incubations of INS-1E cells were only possible for rotenone at 25 mmol·l^−1^ glucose but not for rotenone at 3 mmol·l^−1^ glucose and not for antimycin *A*, since both of the last two conditions profoundly inhibited respiration and induced cell death (Figure 5(b)). Compared to 20 min monitoring with MitoSOX, a different pattern was found when the accumulation of ROS after 2 hr was estimated using the matrix MitoB probe and LC-MS quantification (Figures 7(a) and 7(b)). At 25 mmol·l^−1^ glucose, all of the tested agents except for S3QEL produced a slightly enhanced H_2_O_2_ accumulation, compared to controls with no agents (Figures 7(a) and 7(b)). The matrix-targeted plastoquinone SkQ1 neither decreased nor increased ROS accumulation in INS-1E cells at 3 mmol·l^−1^ glucose (Figure 7(a)). Also, related to the rotenone-induced oxidative stress at 25 mmol·l^−1^ glucose, no tested agents decreased the levels accumulated with rotenone alone (Figure 7(b)). The highest prooxidant effects were found for S1QEL, as was found by monitoring with MitoSOX. Next, we attempted to employ the 4 hr MitoB assay for isolated pancreatic islets. The obtained data correlated better with those obtained from MitoSOX data for INS-1E cells (Figure 8(a)). The better correlation is pointed out by the fact that the islets were resistant to cell death (Figure 8(a)).

### 3.6. Mitochondrial Network Fragmentation Is Prevented by SkQ1

**Figure 9:**
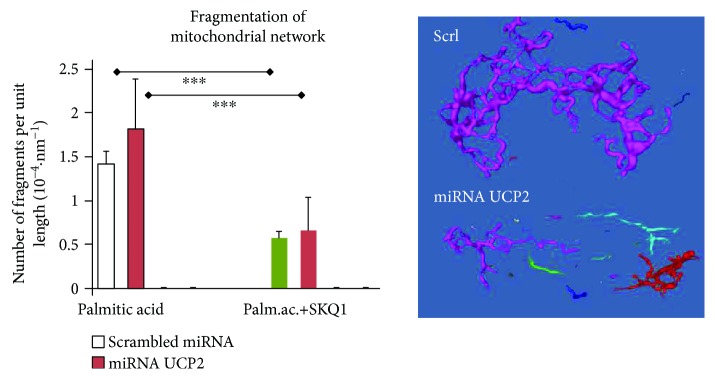
Prevention of palmitic acid-induced mitochondrial network fragmentation in INS-1E cells by SkQ1. 3D superresolution images of the mitochondrial network in mtEos-transfected INS-1E cells preincubated in a medium with 3 mmol·l^−1^ glucose were analyzed after 60 min incubation with 150 *μ*mol·l^−1^ palmitic acid for the number of fragments. Using the Amira Skeleton algorithm (see inset for the resulting mitochondrial network models), the network was artificially connected, and its whole length was taken as a basic parameter. Subsequently, the number of fragments per unit of length was calculated and plotted. Where indicated, 1 nmol·l^−1^ SkQ1 was added at the beginning of a 60 min incubation. The error bars represent the standard error of the mean of 5–10 3D images. ANOVA (*n* = 5–10): ∗∗∗*p* < 0.001.

Besides its antioxidant effects, SkQ1 exhibited also beneficial effects in preventing the excessive fragmentation of the mitochondrial network in the INS-1E cells under specific conditions. Thus, 150 *μ*mol·l^−1^ palmitic acid added to INS-1E cells preincubated with 3 mmol·l^−1^ glucose substantially fragmented the mitochondrial network (cf. Reference [55]), as monitored by 3D PALM superresolution microscopy with the mitochondrial matrix-targeted photoconvertible fluorophore Eos (Figure 9). Note that Eos photoconvertibility with a UV light enables the superresolution imaging mode. UCP2-silenced cells were fragmented similarly. The latter data were not significantly different from the data of cells transfected with scrambled miRNA. SkQ1 at a 1 nmol·l^−1^ concentration was able to prevent more than half of these fragments (Figure 9).

## 4. Discussion

### 4.1. Canonical Antioxidant Effects of Mitochondrial Matrix-Targeted Antioxidants

Mitochondrial matrix-targeted antioxidants have been developed for decades, and a number of compounds have entered the stage of clinical testing. Only a few of them have been registered by national drug testing authorities. For example, SkQ1 was registered in Russia in the form of eye drops to cure dry eye syndrome and is currently under clinical trials in the USA (ClinicalTrials.gov identifier: NCT03764735). Despite the previous preclinical studies, there are still several mechanisms delineated to explain the various effects of SkQ1 on isolated mitochondria and cell cultures. This fact led us to this work to study in detail the dependence of the antioxidant properties of SkQ1 on the superoxide produced by mitochondrial respiratory chain complexes and possible mechanism(s) associated with the activity of the mitochondrial uncoupling protein UCP2. Since recently Brand and colleagues and Wong et al. have developed antioxidants acting within the specific sites of superoxide formation at the complex I or III [45–47], we compared their effects to those observed for SkQ1 to elucidate the involved mechanism(s) in more detail.

The main finding, typical for all tested conditions in this work, is that even for the noncancer cells, such as insulinoma INS-1E cells, the antioxidant efficiency depends strongly on their metabolic activity. We recall that aerobic glycolysis (Warburg phenotype) is absent in INS-1E cells (otherwise, they could not secrete insulin), and ATP predominantly originates from oxidative phosphorylation (OXPHOS) [51].

### 4.2. Antioxidant Effect of SkQ1

The effects observed for SkQ1 resembled those found in cancer cells, hepatocellular carcinoma HepG2 cells [34]. Of course, distinctions in metabolic modes have to be carefully considered. At high glucose, inducing the maximum OXPHOS rate and insulin secretion in INS-1E cells [51], SkQ1 acts as a strong antioxidant and exerts an intermediate antioxidant strength with regard to the oxidative stress simulated by rotenone. The former effect was also confirmed with the isolated pancreatic islets. We also observed that SkQ1 has no effect on the insulin secretion stimulated by glucose (Plecitá-Hlavatá, unpublished data).

SkQ1 has been shown to be able to reaccelerate retarded electron transfer when it is blocked at mitochondrial respiratory chain complex I in HepG2 cells [34]. The effect on INS-1E cells vs. rotenone was similar under conditions of maximum OXPHOS, i.e., at high glucose levels (25 mmol·l^−1^) and was even stronger at low glucose. The pattern in INS-1E cells at 25 mmol·l^−1^ glucose was independent of the presence of UCP2. Consequently, we can conclude that the lower antioxidant ability of SkQ1 observed vs. rotenone at high glucose could originate from a high substrate pressure due to the rotenone block of complex I, as in HepG2 cells [34].

At low glucose levels, simulating fasting conditions *in vivo*, the opposite pattern was found in terms of SkQ1 antioxidant strength. This was more intensive with the rotenone-induced oxidative stress and had a lower strength with no agents added. Despite the fact that the MitoB assay was unable to confirm this pattern for INS-1E cells, SkQ1 and S3QEL, added in the absence of other agents, exhibited a decreased 4 hr ROS accumulation in pancreatic islets at high glucose and virtually unchanged at low glucose levels (for islets 5 mmol·l^−1^).

Moreover, due to the lack of an antioxidant role of SkQ1 upon UCP2 silencing, we demonstrated that part of the antioxidant action of SkQ1 stems from the mechanism, suggested by Skulachev et al. [30]. This might be manifested when a certain level of endogenous fatty acids is present. For the postprandial state of pancreatic *β*-cells, fatty acids derived from lipids in the diet are typically metabolized [2, 3, 20]. Hence, the mechanism is plausible *in vivo*. This mechanism relies on the ion pair of the SkQ1 cation (charged TPP^+^ moiety) with the anionic fatty acid [30]. The resulting {SkQ1^+^and fatty acid anion^−^} penetrates across the membrane, as facilitated by the SLC25 gene family of carrier proteins, such as the ADP/ATP carrier or UCP2, according to Skulachev et al. [30]. Nevertheless, ion pairs are known to penetrate readily across the membrane lipid bilayer; hence, UCP2 may alternatively ensure the uniport of the anionic fatty acid; the ion pair crosses the membrane, and SkQ1^+^ also crosses the membrane (this is the ultimate principle for all mitochondria-targeted compounds). As a result, the cycling is ensured, and the concurrent fatty acid cycling ensures the proton short circuiting leading to the attenuation of superoxide formation (Figure 1(c)).

### 4.3. Antioxidant Effect of Target-Specific Antioxidants

As for the target-specific antioxidants, S1QEL was selected to act at site I_Q_ of superoxide formation (complex I ubiquinone binding site in the vicinity of the rotenone binding site). We confirmed that S1QEL may indeed prevent rotenone-induced oxidative stress. Likewise, S3QEL was selected to act at site III_Qo_ of superoxide formation (the complex III outer ubiquinol binding site in the vicinity of the antimycin *A* binding site). We demonstrated that S3QEL may indeed prevent antimycin *A*-induced oxidative stress. Moreover, as for HepG2 cells [34], we found that S3QEL also attenuates the rotenone-induced oxidative stress. In intact INS-1E cells or pancreatic islets, S3QEL seems to be superior to S1QEL, since it lacks the prooxidant effect (see below).

### 4.4. Intensity of Antioxidant Effect Depends on Metabolic Mode

Moreover, the most important conclusion, derived from the reported findings, suggests that the balance between reduction/oxidation, i.e., between the antioxidant role and the prooxidant regeneration of a given antioxidant, depends on the metabolic mode (see also [34]). There have been previous reports of the successful antioxidant yet incomplete (~60%) action of SkQ1 [34], 65% antioxidant action of MitoQ [28] and ~70% action of S1QEL [34] in relation to the rotenone-induced elevation of superoxide production in glycolytic hepatocellular carcinoma HepG2 cells. However, in rotenone-inhibited aglycemic HepG2 cells, SkQ1 only exhibited a slight (nonsignificant 10%) antioxidant effect [34], whereas MitoQ_10_ had a 20% antioxidant action [28], and S1QEL was highly prooxidant [34]. Note that in the absence of glucose but with galactose, HepG2 cells are forced to oxidative phosphorylation by having glucose-6-phosphate from the galactose metabolism via the Leroi pathway [56]. We have speculated that rotenone-inhibited aglycemic cells have a higher substrate pressure than the glycolytic cells with rotenone. Hence, the high substrate pressure should prevent the regeneration of SkQ1H_2_ [34].

In rotenone-inhibited INS-1E cells, the antioxidant action of SkQ1 had a similar intensity (~60%) to that found in glycolytic HepG2 cells. Also, S3QEL prevented more than 60% of the rotenone-induced superoxide release into the matrix of INS-1E cells (Figure 4(a)). A different pattern was previously reported in cancer cells, compared to the positive antioxidant action of SkQ1 in INS-1E cells at 25 mmol·l^−1^ glucose: SkQ1 had no effect in glycolytic HepG2 cells with 5 mM glucose and in aglycemic HepG2 cells, whereas a prooxidant effect of SkQ1 was found in hyperglycemic HepG2 cells. This is similar to the intensive prooxidant effect of MitoQ_10_ in both glycolytic and aglycemic HepG2 cells [28].

We can compare these reported results to the current finding of a strong antioxidant effect of SkQ1 in INS-1E cells with no agents (Figures 2, 3(a), and 3(b)). S3QEL was able to suppress the superoxide formation of INS-1E cells when monitored within a short time interval (Figure 4(a)) as well as a 4 hr ROS accumulation in pancreatic islets (Figure 8(a)). Islets turned out to be more resistant to cell death during the long-term incubations than INS-1E cells. This may explain why we were unable to observe the antioxidant action of S3QEL vs. a rotenone-induced 2 hr ROS accumulation (Figure 7(b)). Earlier cell death in the particular controls with rotenone alone most likely caused ROS accumulation to also stop earlier, and so, this cannot be used as 100% for normalization. The same is true for SkQ1, the effect of which was most likely also normalized to incorrect “rotenone samples” (Figure 7(a)).

### 4.5. Unintended Prooxidant Role of Antioxidants

The way that the reduced antioxidant is regenerated dictates whether the antioxidant or the prooxidant role will be exhibited. An important case is typical, in which interference from incoming electrons is faster for an antioxidant compound at the given site of the respiratory chain than the regeneration of the reduced antioxidant compound at another site. In this case, the overall effect results in the predominance of the antioxidant effect. Usually this is given by the affinity of the antioxidant to the site where it receives electrons; hence, the electron transfer proceeds more readily. In contrast, when the regeneration (oxidation) of the intended antioxidant is faster (the regeneration site exhibits a high affinity for the antioxidant) than the intended antioxidant reaction, in which electrons are received at a different site, then the prooxidation prevails. Consequently, the expected antioxidant effect ceases. It can be predicted that the relative affinities of the “antioxidant” vs. “regeneration” site may depend on the metabolic mode in certain cases.

Among the three tested mitochondrial matrix-targeted antioxidants, S1QEL did not exhibit a strong antioxidant effect in cells with the rotenone-inhibited complex I. In the intact INS-1E cells, and isolated pancreatic islets, S1QEL even exhibited a prooxidant effect. The latter can be interpreted in terms of the requirement for regeneration of the reduced S1QELH_2_ by oxidation at a currently unknown site [34] and a higher intensity or rate of this regeneration compared to the reduction at the I_Q_ site. This oxidation, as with MitoQ_10_, efficiently slows down the electron transfer via the respiratory chain [28]. As a result, this slowdown creates a prooxidant effect in cells. This effect exceeds the intended antioxidant action within the Q site of complex I, for which S1QEL has been selected from a wide panel of chemical compounds [45].

S1QEL was previously found to be an antioxidant (preventing ~40–50% of superoxide formation) in glycolytic HepG2 cells but a strong prooxidant (8-fold increase in superoxide matrix release) in aglycemic HepG2 cells [34]. The latter can be now compared with the observed slight (10%) prooxidant effect of S1QEL on INS-1E cells at 25 mmol·l^−1^ glucose (Figure 4(a)), where a lower substrate pressure can be expected due to the action of redox shuttles [4]. In contrast, a strong prooxidant effect of S1QEL was found for accumulated ROS in both cells (Figure 7(a)) and isolated pancreatic islets (Figures 8(a) and 8(b)).

### 4.6. Predicted Antioxidant Activity of SkQ1 in Pancreatic *β*-Cells

We can speculate how to translate the obtained data into a use of SkQ1 for the possible prevention of oxidative stress in pancreatic *β*-cells *in vivo* [24, 25]. In accordance with currently available data [30–36] and our basic screening, we can speculatively predict that in intact *β*-cells, SkQ1 might have a beneficial antioxidant role in the postprandial (fed) state, whereas it will not be manifested during fasting. This is analogical to the observed prevention of 4 hr ROS accumulation with high glucose and no prevention with low glucose (Figure 8(a)). We may also speculate that the antioxidant role of SkQ1 can persist (possibly with less intensive effects) when the oxidative stress is established by elevated superoxide formation due to retarded electron transfer in complex I or at the III_Qo_ site of complex III of the respiratory chain [34]. We might therefore reasonably predict that the oxidative stress that frequently originates in pancreatic *β*-cells *in vivo* might be at least somewhat prevented.

However, these predictions should be made with caution due to the dependence of the outcome on the metabolic mode. The established metabolic mode in pancreatic *β*-cells should always include glycolysis tightly coupled to oxidative phosphorylation [1–4] and a lower substrate pressure with high glucose [4]. Despite all of the above, during chronic treatment with SkQ1, a plethora of metabolic modes can occur due to distinct metabolism and related insulin secretion that is stimulated not only by the glucose intake but also by fatty acid intake or branched-chain keto-acid (amino acid) metabolism. Caution should be taken due to the possible prevalence of the {SkQ1^+^and fatty acid anion^−^} cycling mechanism, which could uncouple mitochondria and prevent the mechanism of insulin secretion in pancreatic *β*-cells. As a result, studies of the long-term effects of antioxidants are required under controlled nutrition regime. A major improvement would also be the targeted delivery of SkQ1 to the pancreas or even to pancreatic islets.

### 4.7. Predicted Antioxidant Activity of S3QEL in Pancreatic *β*-Cells

We can also appreciate the ability of S3QEL to be an antioxidant in terms of complex I-related as well as complex III site III_Qo_-related oxidative stress in both HepG2 cells of all metabolic modes [34] as well as in INS1-E cells (Figure 4(a)) and islets (Figure 8(a)). In this respect, S3QEL may be predicted to be a suitable antioxidant for pancreatic *β*-cells with ongoing oxidative stress, unless unwanted effects are found in other tissues, e.g., due to interference with redox signaling. For example, it might decrease or suppress the redox signaling occurring during the hypoxic redox initiation of the proline hydroxylase domain containing enzymes (PHDs). Oxidation of their iron^II^ initiates HIF-1*α* stabilization and transcriptome reprogramming in hypoxia [17, 56, 57]. However, no such action of S3QEL has been reported yet.

### 4.8. Mitochondrial Network Fragmentation as a Link between Oxidative Stress and Mitophagy and Apoptosis

We have encountered an interesting phenomenon of the SkQ1-mediated prevention of fatty acid-induced (probably uncoupling-induced) fragmentation, i.e., fission, of the mitochondrial network. Previous research indicated that fatty acid-induced mitochondrial fission may not always lead to mitochondrial dysfunction [55]. Physiological levels of fission of the mitochondrial network are required for housekeeping by mitochondria-specific autophagy (mitophagy) [58]. In contrast, excessive fission can lead to apoptosis in pancreatic *β*-cells [59]. The {SkQ1^+^ & fatty acid anion^−^} cycling mechanism may outcompete the uncoupling initiating the inhibition of mitochondrial network fusion due to the cleavage of OPA1 [60]. Alternatively, SkQ1 by its antioxidant action attenuates a putative mitochondrial redox signal leading to fission as well. We currently cannot distinguish between these two possibilities.

## 5. Conclusions

We demonstrate for the first time the antioxidant properties of SkQ1 and S3QEL in pancreatic *β*-cells. A profound short-term antioxidant role was found in model pancreatic *β*-cells, INS-1E cells. In parallel, we demonstrated an antioxidant action in the form of the 4 hr prevention of ROS accumulation in isolated mouse pancreatic islets. However, their antioxidant action was manifested mostly with high glucose, simulating the fed state *in vivo*. The dependence on the cell metabolic mode was explained by affecting the relative affinities of the “antioxidant” vs. “regeneration” site for the antioxidant compound. When the latter predominates, the antioxidant effect ceases. SkQ1 and S3QEL may be suggested for preclinical trials in diabetic mouse or rat models.

## Data Availability

The data used to support the findings of this study are available from the corresponding author upon request.
